# Melanotic Xp11 translocation renal cancer: a report of a distinctive case and a review of the literature

**DOI:** 10.1186/s13000-018-0731-y

**Published:** 2018-08-13

**Authors:** Hongbiao Jing, Hong Wei, Hongtu Yuan, Yahong Li, Ning Li, Dianbin Mu

**Affiliations:** 1 Department of Pathology, Shandong Cancer Hospital Affiliated to Shandong University, No. 440 Jiyan Road, Jinan, 250117 China; 2 The Sixth People Hospital of Jinan City, No. 308 Huiquan Road, Zhangqiu, 250200 China; 3 Guangzhou LBP Medical Technology Co., Ltd, No. 11 Nanxiang Third Road, Guangzhou, 510663 China

**Keywords:** Kidney, Melanin, Neoplasm, *TFE3*, Translocation, Xp11

## Abstract

**Background:**

Melanotic Xp11 translocation renal cancer (TRC) is a newly described exceedingly rare tumor, and its characterization remains controversial. This study aimed to describe a case of distinctive melanotic Xp11 TRC and to elucidate its clinicopathological and molecular genetic features.

**Case presentation:**

A 44-year-old Chinese female presented with a left renal mass. Abdominal ultrasonography and computed tomography (CT) scans revealed a 4.5 cm × 4.0 cm mass in the left kidney. Grossly, the well-demarcated mass was black with moderately firm consistency. Microscopic examination indicated that the tumor was characterized by the presence of nests and cords of polygonal cells with clear and granular eosinophilic cytoplasm, central round to oval nuclei and occasional nucleoli. Intracytoplasmic melanin was observed in approximately 45% of tumor cells. Uniquely, the tumor presented with intranuclear eosinophilic pseudoinclusions and thick-walled stromal blood vessels. IHC showed that tumor cells were diffusely positive for TFE3 and exhibited patchy and weak HMB45 staining. FISH confirmed the presence of *TFE3* rearrangement.

**Conclusion:**

This case is the twentieth published case of melanotic Xp11 TRC. Moreover, the present patient had a favorable prognosis given that she was disease free at her 113-month postoperative follow-up. Our case adds to the small body of literature on these exceptionally rare tumors and widens their clinicopathological spectrum.

## Background

Melanotic Xp11 translocation renal cancer (TRC) is a newly defined entity that is characterized by sheets and nests of epithelioid neoplastic cells with clear to finely granular eosinophilic cytoplasm and intracytoplasmic melanin; immunohistochemical positivity for melanocytic markers (such as HMB45 and Melan A) but negativity for epithelial and muscular markers; and rearrangement of the transcription factor enhancer 3 (*TFE3*) gene. Since it was first described by Argani et al. in 2009, only 19 cases of melanotic Xp11 TRC have been documented in English-language publications (Table 1) [1–11]. In this study, we report an additional case of melanotic Xp11 TRC with distinctive clinicopathological features. A review of the literature was performed to elucidate the clinicopathological and molecular genetic features of this rare tumor.

**Table 1:** Clinicopathological data for 19 reported cases of Xp11 TRC

Sex/Age (Yr)	Location	Size(cm)	Treatment	Outcome (Mo)	Reference
M/11	L kidney	21.5	Nephrectomy	NA	1
F/12	L kidney	NA	Biopsy	DOD/9	1
F/18	R kidney	9.6	Nephroureterectomy	NED/3	2
F/30	Kidney	12.5	Nephrectomy	NA	3
F/15	R ovary	10.5	Tumorectomy	NED/9	4
F/14	Kidney	NA	NA	NA	5
F/34	L kidney	4.8	Partial nephrectomy	NED/22	6
F/46	R kidney	5.8	Nephrectomy	DOD/24	7
M/35	R kidney	7.0	Nephrectomy	DOD/18	7
F/38	L kidney	4.0	Nephrectomy	NA	7
F/44	L kidney	4.5	Partial nephrectomy	NA	7
F/17	L kidney	5.0	Nephrectomy	NA	7
M/25	L kidney	4.0	Nephrectomy	NA	7
M/15	L kidney	5.5	Nephrectomy	NA	7
M/34	Kidney	9.7	NA	NA	8
F/21	Kidney	NA	NA	NA	8
F/18	R kidney	21.5	Nephrectomy	Recent case	9
F/36	L kidney	5.2	Nephrectomy	NED/6	10
M/36	L kidney	6.0	Nephrectomy	NED/84	11
F/44	L kidney	4.5	Nephrectomy	NED/113	Present case

Abbreviations: *TRC* translocation renal cancer; *Yr* year; *Mo* month; *M* male; *L* left; *NA* not available; *F* female; *DOD* dead of disease; *R* right; *NED* no evidence of disease

## Case presentation

A 44-year-old Chinese female presented with a left renal mass that had been incidentally discovered on ultrasonography during a health check-up. She had no history of flank pain, gross hematuria, foamy urine, pyuria, dysuria, frequent urination, painful urination, urgent urination, or weight loss. Her past medical history and family history were unremarkable. A physical examination produced negative results for the lumbar zones. Routine laboratory test data were within normal limits. Abdominal ultrasonography revealed a 4.5 cm × 4.0 cm nodular solid mass with calcifications of heterogeneous density in the lower portion of the left kidney. The tumor was hypervascular and exhibited a massive internal hyperechoic area. An abdominal CT scan also confirmed a well-circumscribed calcified renal mass. No lymphadenopathy or ascites was discovered. The patient underwent a right radical nephrectomy and partial ureterectomy. At laparotomy, no gross evidence of metastatic spread or the involvement of other intra-abdominal organs was observed. The patient’s postoperative course was uneventful. She refused additional treatment, including radiotherapy or chemotherapy, except for postoperative surveillance with CT. At present, 113 months after surgery, the patient remains well, with no evidence of recurrence or metastasis.

**Fig. 1:**
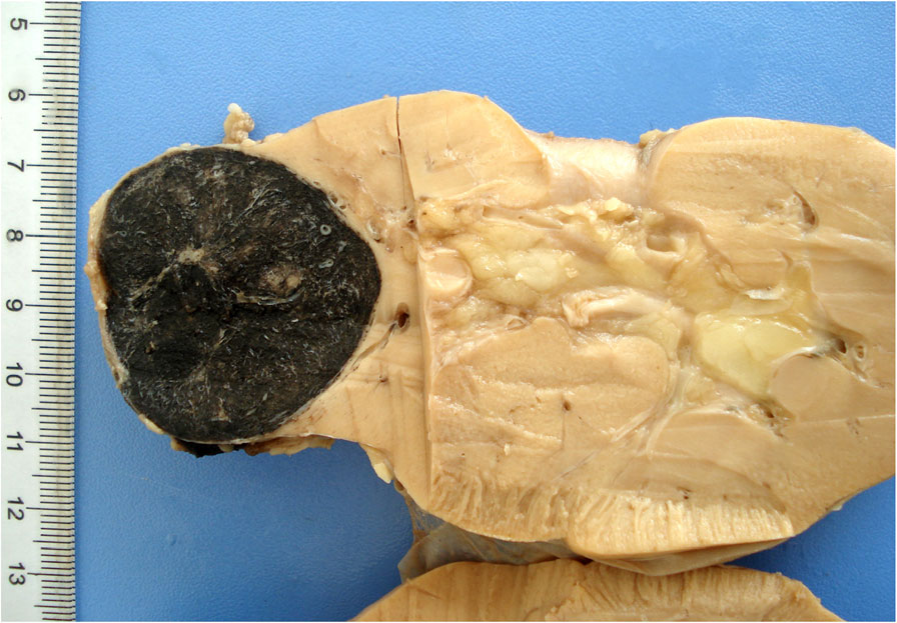
The well-defined tumor exhibited black pigment throughout the lesion on the cut surface

On macroscopic examination, the non-encapsulated nodular mass, sized 4.5 cm × 3.5 cm × 3.0 cm, was located in the inferior pole of the kidney. It was well defined and black in color with moderately firm consistency (Fig. 1). The lesion extended to but not through the renal capsule.

**Fig. 2:**
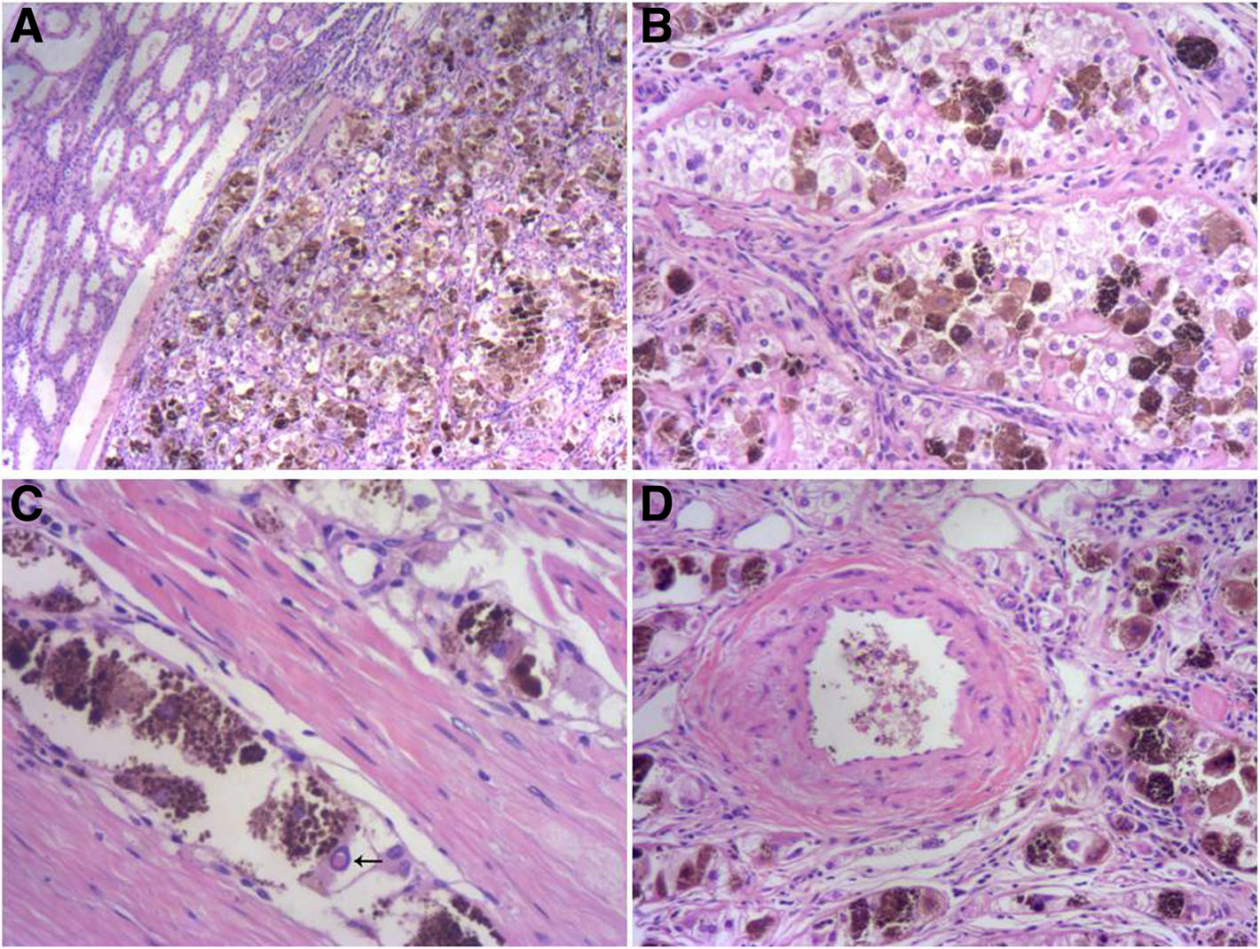
**a** The non-encapsulated tumor was sharply demarcated from the adjacent renal parenchyma (H&E staining, × 40). **b** The neoplasm consisted of polygonal tumor cells with clear and granular cytoplasm arranged in nests and cords delimited by thin-walled fibrovascular septa as well as numerous neoplastic cells containing intracytoplasmic brown pigment that were found throughout the lesion (H&E staining, × 100). **c** Occasional nuclear pseudoinclusions (arrow) were present (H&E staining, × 200). **d** Thick-walled blood vessels were observed(H&E staining, × 100)

**Fig. 3:**
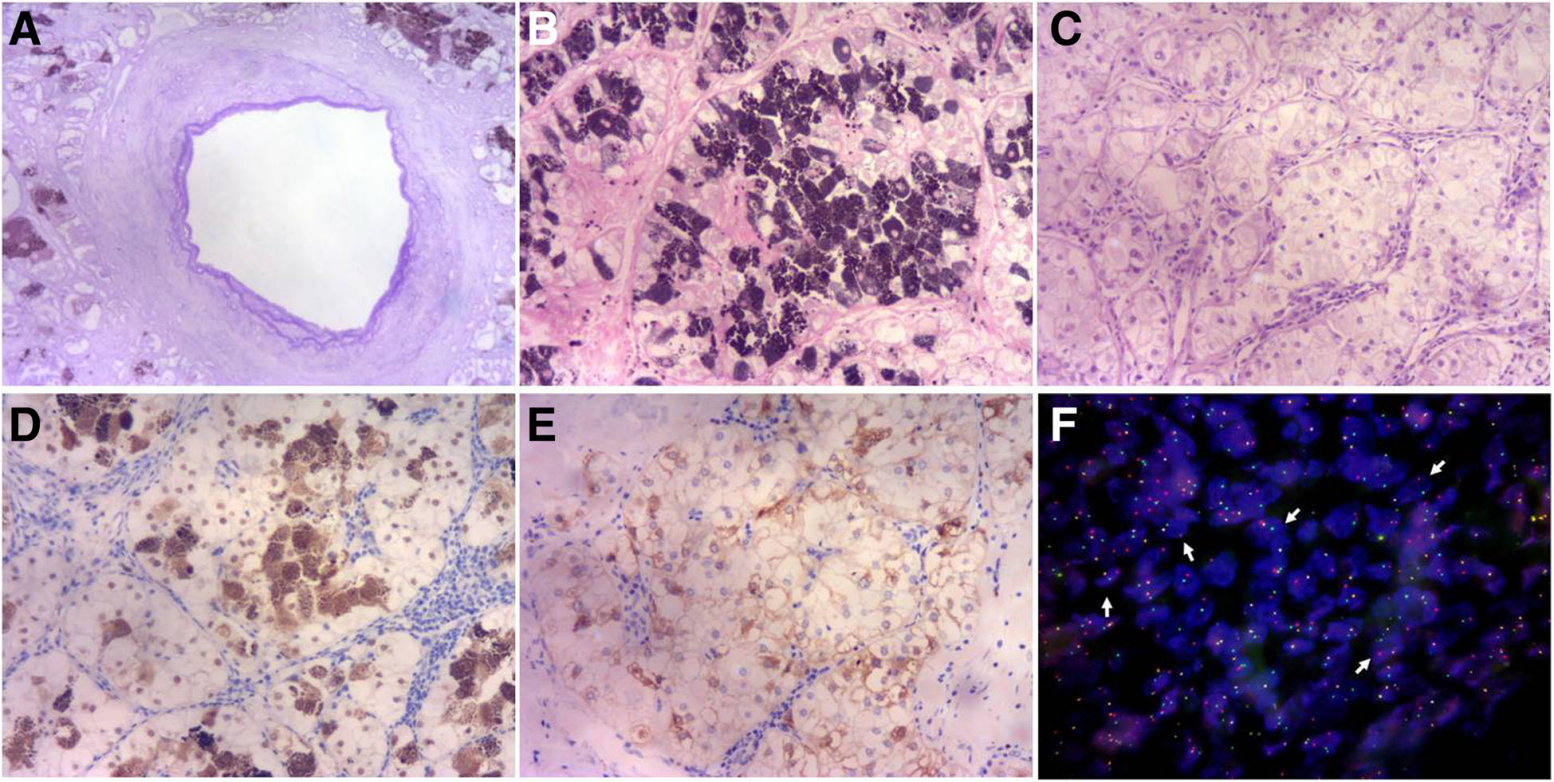
**a** The elastic fibers of thick-walled blood vessels are highlighted by Gomrori’s aldehyde-fuchsin staining (× 100). The cytoplasmic pigment is positive for Fontana-Masson staining (**b**) (× 100); it was removed by potassium permanganate bleaching (**c**) (× 100). Tumor cells exhibit diffuse nuclear immunoreactivity for TFE3 (**d**) (IHC staining, × 100) and patchy cytoplasmic positivity for HMB45 (**e**) (IHC staining, × 100). **f** Break-apart FISH for *TFE3* produced normal fused signals and split signals (arrows); one pair of each type of signal is depicted (× 1000)

With the exception of abundant intracytoplasmic pigmentation, the lesion’s histological features were consistent with those of a clear cell renal cell carcinoma. Low-power observations indicated that the tumor was well demarcated from the renal parenchyma; lacked a fibrous capsule; and was composed of nests and cords of polygonal tumor cells, predominantly nests, and intervening delicate thin-walled fibrovascular septa (Fig. 2a). Cells in certain nests were focally discohesive, resulting in an alveolar structure. On high-power examination, approximately 55% of tumor cells contained abundant, clear and finely granular eosinophilic cytoplasm and distinct cell borders. Characteristically, the remaining 45% of tumor cells presented with variable quantities of intracytoplasmic brown pigment, ranging from finely dispersed small cytoplasmic granules to massive agglomerations (Fig. 2b). Tumor cells’ central round to oval nuclei had evenly distributed chromatin with occasional small, non-prominent nucleoli. Intranuclear eosinophilic cytoplasmic pseudoinclusions, which are exceedingly rare, were also present (Fig. 2c). There was inconspicuous nuclear pleomorphism, and the tumor was assigned a low nuclear grade. Mitotic figures were extremely uncommon. Intriguingly, scattered thick-walled blood vessels with the normal elastic content of arteries, as demonstrated via Gomori’s aldehyde-fuchsin staining, and unusual focal eccentric hyalinization were present throughout the tumor (Figs. 2d and 3a). In addition, calcifications were readily observed and had frequently formed on hyaline nodules. Neither necrosis nor hemorrhage was observed. Histochemical staining analyses indicated that the brown pigment was negative in Prussian blue staining but was highlighted by Fontana-Masson staining and was completely bleached by potassium permanganate; these findings suggested that this pigment was melanin (Fig. 3b and c). Immunohistochemical staining revealed strong and diffuse nuclear staining for TFE3 in the tumor cells (Dako, Carpinteria, CA, USA, 1:800) (Fig. 3d), which was performed as previously described [12]. Patchy and weak cytoplasmic staining for HMB45 (Dako, 1:500) was also observed (Fig. 3e). Ki-67 (Dako, 1:800) stained only approximately 3% of tumor cells (based on 1000 cells counted using Image-Pro Plus Version 5.1 C (Media Cybernetics, Silver Spring, MD, USA)). In contrast, staining for all other assessed immunomarkers, including AE1/AE3 (Dako, 1:1000), epithelial membrane antigen (EMA; Dako, 1:50), CK7 (Dako, 1:400), CK20 (Dako, 1:100), vimentin (Neomarkers, Fremont, CA, USA, 1:500), smooth muscle actin (SMA; Neomarkers, 1:800), desmin (Dako, 1:400), CD10 (Neomarkers, 1:200), PAX8 (ProteinTech Group, Chicago, IL, USA, 1:200), renal cell carcinoma (RCC) marker (Vector, Burlingame, CA, USA, 1:200), Cathepsin K (Abcam, Cambridge, MA, USA, 1:800), Melan A (Dako, 1:400), MiTF (Dako, 1:150), S100 protein (Dako, 1:1000), CD117 (Dako, 1:600), CD34 (Dako, 1:200), and DOG-1 (Santa Cruz Biotechnology, Santa Cruz, CA, USA, 1:400), was negative.

Break-apart FISH analysis was performed to detect TFE3 gene fusion in paraffin-embedded tissues with a probe consisting of 2 contigs that flank the TFE3 gene on Xp11.2 as previously described [13]. The distal contig consists of 3 bacterial artificial chromosomes (BAC) clones (RP11-344 N17, CTD-3028 J4, and CTD2522M13) labeled with Spectrum Orange, and the proximal contig consists of 2 BAC clones (RP11-57A11 and RP11211H10) labeled with Spectrum Green; from BacPac Resources at the Children’s Hospital Oakland Research Institute and Invitrogen. FISH analysis revealed the presence of a *TFE3* rearrangement (Fig. 3f). The tumor was diagnosed as Xp11 TRC and staged as T1a.

## Discussion

A melanotic Xp11 TRC, which is a newly discovered neoplastic entity described by Argani et al., is a rare renal tumor with distinct histologic, immunohistochemical, and molecular genetic features [1]. It was originally postulated by Argani et al. to be most closely related to Xp11 translocation perivascular epithelioid cell neoplasm (PEComa), and this has subsequently been supported [1, 8]. Histologically, this lesion characteristically consists of sheets and nests of epithelioid cells with clear to finely granular eosinophilic cytoplasm delimited by delicate capillary vasculature. Notably, a subset of neoplastic cells bear intracytoplasmic melanin pigment. In immunohistochemical analyses, tumor cells exhibit diffuse nuclear labeling for TFE3 protein and cytoplasmic reactivity with Cathepsin K and HMB45. Molecular genetic analysis indicated *TFE3* gene rearrangement [1–11]. The pathologic and genetic features of the renal mass found in our patient satisfied the diagnostic criteria for melanotic Xp11 TRC. To our knowledge, our case is only the twentieth pathologically and genetically confirmed case of this entity to date (Table 1). In addition, among the 20 reported patients with melanotic Xp11 TRC, the present patient was disease free at the longest follow-up (113 months).

As documented in Table 1, melanotic Xp11 TRC generally occurs in young adults (age range: 11–44 years; mean: 27.2 years). The sex ratio among patients with this disease is 6 males to 14 females, revealing a female predilection, albeit with a limited sample size. In cases for which information regarding lesion size is available, lesions have ranged from 4.0 to 21.5 cm (mean: 8.3 cm). Three of nine patients with available follow-up data died due to the tumor. Although melanotic Xp11 TRC lesions were generally in the kidneys, one tumor in an ovary was reported, suggesting that this entity could arise in non-renal locations. As a result, LeGallo et al. chose “melanotic Xp11 neoplasm” as their designation of this entity [4].

The present case highlights three unique clinicopathological aspects of melanotic Xp11 TRC. First, among the 20 documented patients with melanotic Xp11 TRC, the present patient was disease free at the longest follow-up (113 months), suggesting that this tumor has an indolent clinical course. Second, occasional intranuclear eosinophilic cytoplasmic pseudoinclusions of neoplastic cells were detected in this patient’s tumor. Finally, massive thick-walled blood vessels were observed. In contrast to those found in conventional angiomyolipoma (AML), thick-walled vessels in the current lesion exhibited normal elastic fibers and focal eccentric hyalinization. To our knowledge, these three unique features of our case have not been described in the 19 previously reported cases of melanotic Xp11 TRC;therefore, this case widens this entity’s clinicopathological spectrum.

Given the location and pathological appearance of the type of tumor observed in this case, the main differential diagnoses considered should include Xp11 translocation renal cell carcinoma, malignant melanoma, and PEComa, including AML and its epithelioid variant. In fact, the initial clinical and pathological diagnosis for our case was Xp11 translocation renal cell carcinoma due to the tumor’s renal location and histological features of sheets and nests of epithelioid cells with clear to finely granular eosinophilic cytoplasm and intervening delicate fibrovascular septa. However, immunohistochemical negativity for epithelial markers does not exclude Xp11 translocation renal cell carcinoma. Xp11 translocation cell carcinomas are positive for renal tubular markers (including CD10, RCC marker, PAX2, and PAX8) at varying levels [14, 15]. In contrast, melanotic Xp11 TRCs are negative for both epithelial and renal tubular markers [1]. Therefore, the present tumor was determined to be a melanotic TRC rather than an Xp11 translocation renal cell carcinoma based on its histological and immunohistochemical features. The obvious cytoplasmic melanin and immunoreactivity for HMB45 in this tumor could suggest a diagnosis of malignant melanoma. However, malignant melanoma could be excluded because tumor cells of XP11 TRC were negative for S100 protein and MiTF. Moreover, our case involved the complete absence of morphological indications of spindle neoplastic cell differentiation and the presence of nuclear labeling of TFE3 in immunohistochemical analysis; these features are not typical of malignant melanoma [16, 17]. The differentiation of an AML, particularly an epithelioid AML, from a melanotic Xp11 TRC is mainly based on the former tumor’s immunoreactivity for MiTF, a muscle marker (SMA) and melanocytic markers (HMB45 and Melan A) and the latter tumor’s immunohistochemical negativity for MiTF and SMA and positivity for HMB45 in a patchy pattern. In addition, spindle cell components that would favor AML were not observed in the present case. Finally, thick-walled blood vessels in AML lack elastic fibers; in contrast, in the present case, elastic materials were present in such vessels, as demonstrated by histochemical staining [18].

## Conclusion

We report a case of melanotic Xp11 TRC with unique histologic features in a 44-year-old Chinese female. This case adds to the small body of literature on these exceptionally rare tumors and widens their clinicopathological spectrum. Further studies are required to determine the precise clinicopathologic and genetic features of such tumors.

## Abbreviations

BAC: Bacterial artificial chromosome
CT: Computed tomography
FISH: Fluorescence in situ hybridization
H&E: Hematoxylin and eosin
IHC: Immunohistochemistry
PEComa: Perivascular epithelioid cell neoplasm
*TFE3*: Transcription factor enhancer 3
TRC: Translocation renal cancer
